# Polar labeling: silver standard algorithm for training disease classifiers

**DOI:** 10.1093/bioinformatics/btaa088

**Published:** 2020-02-12

**Authors:** Kavishwar B Wagholikar, Hossein Estiri, Marykate Murphy, Shawn N Murphy

**Affiliations:** b1 Laboratory of Computer Science, Massachusetts General Hospital, Boston, MA 02114, USA; b2 Partners Healthcare, Somerville, MA 02145, USA

## Abstract

**Motivation:**

Expert-labeled data are essential to train phenotyping algorithms for cohort identification. However expert labeling is time and labor intensive, and the costs remain prohibitive for scaling phenotyping to wider use-cases.

**Results:**

We present an approach referred to as polar labeling (PL), to create silver standard for training machine learning (ML) for disease classification. We test the hypothesis that ML models trained on the silver standard created by applying PL on unlabeled patient records, are comparable in performance to the ML models trained on gold standard, created by clinical experts through manual review of patient records. We perform experimental validation using health records of 38 023 patients spanning six diseases. Our results demonstrate the superior performance of the proposed approach.

**Availability and implementation:**

We provide a Python implementation of the algorithm and the Python code developed for this study on Github.

**Supplementary information:**

Supplementary data are available at *Bioinformatics* online.

## 1 Introduction

**Fig. 1.**
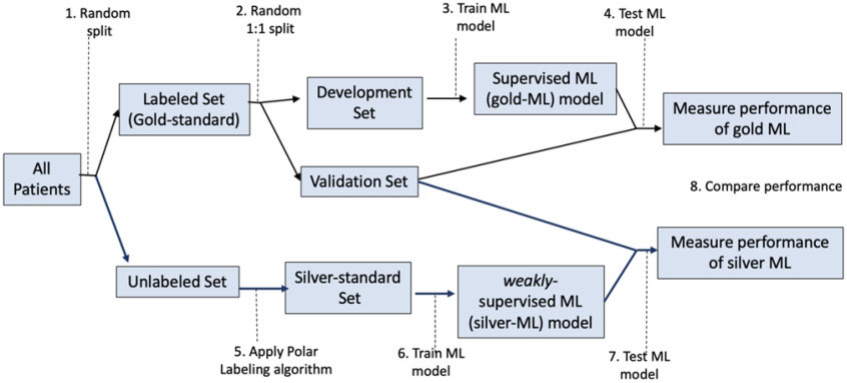
Experimental design

Phenotyping forms the basis of utilizing statistical or machine learning (ML) for research and clinical workflows (Shivade *et al.*, 2014; Xu *et al.*, 2015). However, data in electronic health records (EHR) are considered noisy for training ML algorithms (Hripcsak and Albers, 2013). To address this gap, expert labeling of the data is widely used to train phenotyping algorithms (see Fig. 1; Geraci *et al.*, 2017; Kagawa *et al.*, 2017; Wood *et al.*, 2015). However expert labeling is time and labor intensive, and the costs are prohibitive for scaling phenotyping to wider use-cases (Richesson *et al.*, 2013, 2017) The lack of data quality is considered as the primary obstacle for utilization of AI in healthcare (Hripcsak and Albers, 2013; Jason, The Mitre Corporation, 2017).

Several approaches have been investigated to avert the high costs of expert labeling. Methods to discover optimal inference algorithms, as well as feature selection and feature engineering techniques to combine different data modalities have been shown to improve performance of supervised ML (Carroll *et al.*, 2011; Huang *et al.*, 2004; Teixeira *et al.*, 2017). These approaches serve to minimize the size of required expert labels. Active learning has been observed to be similarly useful in reducing the labeling effort (Chen *et al.*, 2013). Unsupervised learning-based tensor factorization techniques called Limestone and Marble are able to generate phenotype clusters with no predefined phenotype definitions but require manual validation by experts (Ho *et al.*, 2014a, b).

On the other hand, several weakly supervised learning approaches have been investigated. Yu *et al.* (2015) described an automated feature-selection algorithm (AFEP) that uses medical knowledge sources. For rheumatoid arthritis and coronary artery disease cohorts, models trained using the features selected by AFEP achieved comparable or slightly higher accuracy than those trained with exper-curated features. Yu *et al.* improved upon AFEP to develop Surrogate-assisted feature extraction (SAFE). SAFE refined the feature selection using high coefficients of variables generated in ML models trained on a silver standard that was derived from frequency distribution of diagnostic codes (Yu *et al.*, 2017). The performance of algorithms trained using features identified by SAFE was significantly higher than that of those trained on expert curated features for four disease cohorts. However, this approach is complex to implement in contrast to the XPRESS framework developed by Aggarwal *et al.* (2016), in which the silver standard labels are generated by searching disease-related anchor terms in clinical notes. Models trained on this silver standard were reported to be comparable to rule-based models for heart attack and type 2 diabetes mellitus. Furthermore, the XPRESS framework has been implemented as a R software (R Foundation for Statistical Computing, Vienna, Austria) package by Banda and is widely used in the Observational Medical Outcomes Partnership (OMOP) consortium. Deriving from XPRESS, Yu *et al.* (2017) developed Phenorm, which includes SAFE for feature selection but trains ML directly on silver standard labels from frequency distribution of diagnoses, and applies self-regression to incorporate related features for refining the silver standard. However, a downside of this approach is that it involves multiple steps and parameters, and is not available as an opensource implementation. Consequently Phenorm is difficult to implement or replicate by other researchers.

In this paper, we detail our approach for creating a silver standard to bypass the effort for creating human annotation for training ML models. Ours is a weakly supervised learning approach, (Zhou, 2018) that leverages the intuitive concept that even though health data are noisy, patients who are repeatedly ascribed to particular categories are more likely to have true membership of that category. For example, patients with a large number of visits with documentation of codes for asthma are more likely to truly have asthma than those patients who have a lesser number of such codes in comparison. In other words, patients near the high pole of asthma distribution counts will tend to truly suffer from asthma. Our main contribution is that we describe an algorithm implementing this notion, which we refer to as polar labeling (PL) algorithm. Furthermore, we perform experimental validation using expert annotations on patient records for six diseases. Specifically, we test the hypothesis that ML models trained on silver standard (created by applying PL on unlabeled patient records), are comparable in performance to the ML models trained on gold standard, created by clinical experts through manual review of patient records.

We provide a Python implementation of the algorithm and the Python code developed for this study on Github. We hope that our results and source code will enable the research community to further study and use the proposed approach.

## 2 Materials and methods

The experiment described in this paper was carried out on health records of 38 023 patients at Partners Healthcare in Boston who had consented for inclusion of their health records into the institutional biobank. The study was approved by the institutional review board. The patient records were obtained by querying the institutional research patient data repository (Nalichowski *et al.*, 2006; Wattanasin *et al.*, 2008). For conducting this study, we selected six diseases of varying prevalence including asthma, breast cancer, chronic obstructive pulmonary disease (COPD), depression, epilepsy and hypertension. See Supplementary Appendix B for details on disease prevalence.

### 2.1 Experimental design

Figure 1 outlines the experimental design. For each of the chosen diseases, we selected patients from the study dataset, to create a gold-standard set labeled by a clinical expert. The unselected patients formed the unlabeled set (Step 1 in Fig. 1). The gold-standard set was further split randomly and equally into a development set and a validation set (Step 2). A silver-standard set was created by applying the PL algorithm to the unlabeled set.

Development set (Step 3), and the silver-standard set (Step 6) were used to train ML models. To facilitate readability, we henceforth refer to the models as gold-ML and silver-ML, respectively. The validation set was used to measure the performance of both of these models (Steps 4, 7 and 8).

Creation of the validation set was repeated 15 times. In each of the runs, new gold-ML and silver-ML models were trained on the development set and silver-standard set respectively, and then scored on the validation set. These multiple runs were essential to average the performance scores.

What follows is the detailed methodology for (i) creation of gold standard, (ii) creation of silver standard, (iii) PL algorithm, (iv) feature extraction, (v) training of ML models on the development-set and silver standards and (vi) comparison of the performance of the models on the validation set.

### 2.2 Gold standard creation

Samples of 540 patients were drawn from the study dataset for each of the six diseases. Each of the sample consisted of 540 randomly selected patients, such that 100 of these patients had an International Classification of Diseases, ninth revision (ICD-9) code for the disease in question and 365 had at least one other disease. The remaining 75 patients were selected completely at random. For each of these samples, a single clinical expert (nurse practitioner) performed a chart review to annotate the presence of the disease. This expert labeled the patients with one of four classes: Y: present, N: Absent, P: possible, U: unknown/can’t say. The criteria for the disease annotations are provided in Supplementary Appendix A. For our experiments we included only the Y and N labels, which provided more certainty (Table 1).

**Table 1.** Distribution of expert annotations for the six disease cohorts

	Human/expert annotations					Estimated prevalence in USA (%)
Cohort	N	P	U	Y	Total	
Asthma	446	10	3	81	540	7.9
Breast cancer	463	10	3	64	540	0.5
COPD	468	30	3	39	540	4 – 9
Depression	383	9	5	143	540	8.1
Epilepsy	479	5	4	52	540	1.2
Hypertension	222	8	3	307	540	29

Y, present; N, absent; P, possible; U, unknown/can’t say.

### 2.3 Silver-standard creation

The silver-standard is generated from the unlabeled set (Step 5) by applying the PL algorithm.

### 2.4 PL algorithm

**Fig. 2.**
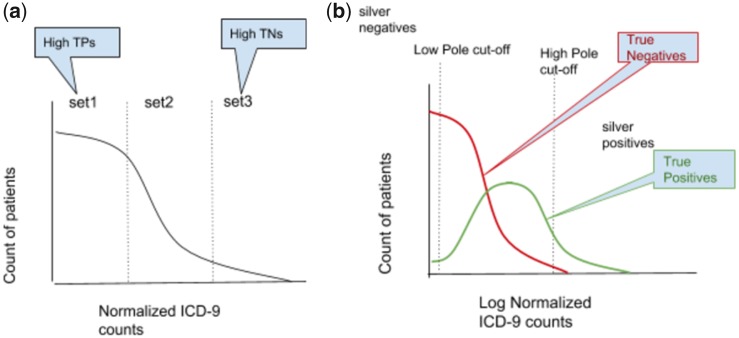
(**a**) Counts of ICD-9 codes follow a log normal distribution. (**b**) Patients to left-side of low-pole are silver negatives and right-side of high pole are silver positives

The steps are elaborated as follows (see Fig. 2):

1. Obtain diagnostic billing counts: The total number of ICD-9 counts that each patient has forms the input for the algorithm.
2. Distribution of log normalized counts: As the distribution shows logarithmic distribution the counts are normalized to log normal form log (*x* + 1). Note that the addition of 1 to the count obviates computation of log for zero counts.
3. Identify ‘potential silver positives’: Patients with log(count+1) > 0 i.e. count > =1 are potential silver positives.
4. Cut-off (alpha) for delineating high pole: The mean and standard deviation for the log normal distribution for ‘potential silver positives’ is computed, and for an arbitrary factor alpha, the ‘high pole cut-off’ is computed as mean + (cut-off parameter *x* standard deviation). The lower pole cut-off is always at 1.0.
5. Silver set: Patients on and to the right of the high pole cut-off are labeled as ‘silver positive’, and patients on and to left of ‘low pole cut-off’ are labeled as ‘silver negative’. Note that all patient with log(count + 1) = 0 i.e. count = 0 are silver negatives.
6. Balance negative silver set: The ‘silver negative’ set is reduced to a smaller subset of randomly selected y patients, where *y* is the size of silver set *x* (size of silver positive/size of potential silver positive). This step ensures that relative distribution of positive and negative cases in the ‘balanced silver set’ is close to that in the entire dataset.
7. Create ‘balanced silver’ set as a union of the ‘positive silver’ set and the ‘balanced negative silver’ set.
8. Output a random sample of the balanced silver set, by creating a random subset of a specified ‘train size’ parameter.

Hence, there are two parameters for the PL algorithm: cut-off parameter (Step 4) and train size parameter (Step 8).

### 2.5 Feature extraction

For applying ML to the dataset, we transformed the health records for each patient to a feature vector, as follows. Clinical experts hand-picked concepts that are relevant for the six diseases, including diagnosis, medications, laboratory results and procedures (Supplementary Appendix C). We computed the total number of times each of these concepts occurs in the health record of a patient, to construct a count vector of the features (*n* = 175) for the patient. For example, PRC_ERVisit and DX_COPD indicate the number of emergency room visits and number of times the diagnosis of COPD was made in the patients record, respectively. The feature vectors were then used for training and scoring the ML algorithm.

### 2.6 ML model training

The gold and silver ML models were trained on the development set (part of gold standard) and silver-standard, respectively. We trained two types of ML models—random forest (RF) and logistic regression (LR). The RF classifiers were trained using 1000 estimators, using the Gini score to split the trees. The LR model was fit with L2 regularization, with maximum threshold of 10 000 iterations for optimization. The coefficient of regularization was selected by performing 5-fold cross-validation using the area under the receiver operating characteristics (AUROC) curve for scoring. The developed models were output in Steps 3 and 6 in Figure 1.

### 2.7 Performance comparison

We compared the AUROC of the gold and silver ML models (Fig. 4 and Table 2). T-test was used to determine the significance of the difference in *P*-values, with a *P*-value of 0.05 acting as a cut-off for statistical significance. For baseline, we measured the AUROC for the simple rule of predicting a disease for a patient if the diagnosis code for the disease is present in the patient’s health record.

### 2.8 Opensource code

We have included a Github link to our Python implementation of the PL algorithm, along with the code for carrying out the experiment described in this paper, which includes a dummy dataset. In addition, a live Jupyter-python notebook for conducting the experiment is available as mybinder link.

## 3 Results

**Fig. 3.**
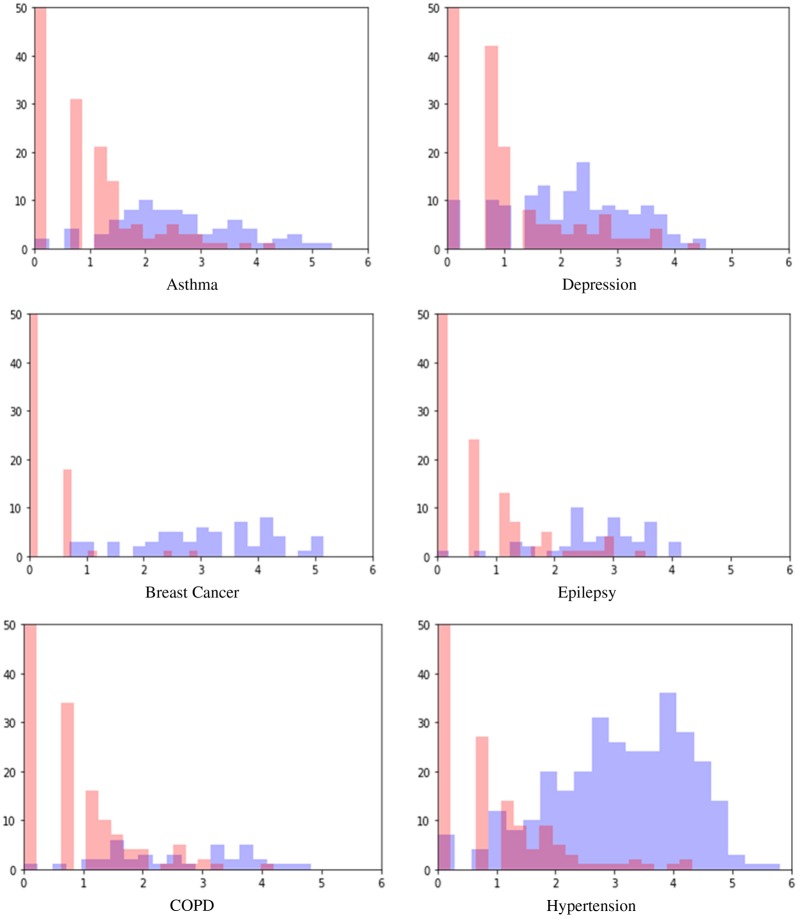
Log-normal distribution of counts of billing codes, using the labeled dataset, shows distinct but overlapping log-normal distribution for both true-positive and true-negative patients. The poles of the distributions show areas of enriched true-positive (blue colored bars)/true-negative (red colored bars) patients. The *y*-axis is the count of patients and the *x*-axis is the log of diagnostic ICD-9 count. (Color version of this figure is available at *Bioinformatics* online.)

The PL algorithm for the creation of silver standard leverages the intuitive concept that even though medical data are noisy, patients that are repeatedly ascribed to particular categories are more likely to hold true membership of that category. Figure 3 demonstrates this concept on the labeled dataset.

**Fig. 4.**
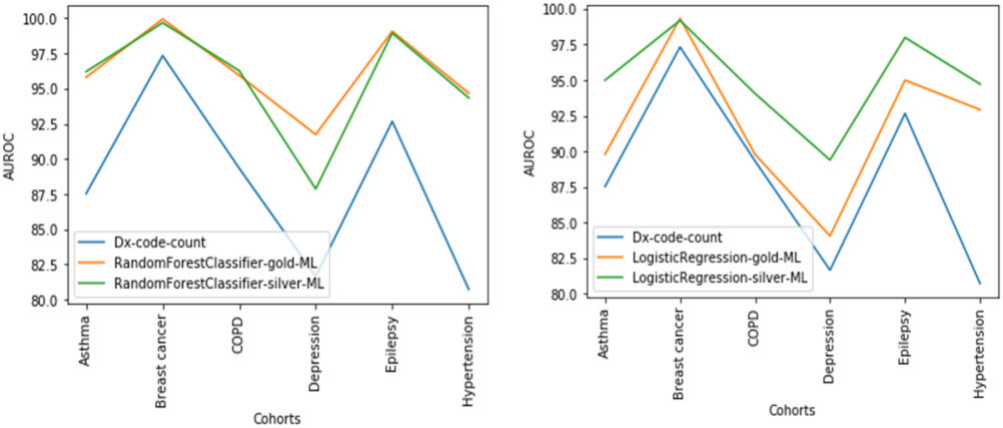
AUROC for the phenotyping models on the validation sets. The suffix of gold-ML refers to the ML models trained on the ‘gold-standard development set’, and silver-ML refers to the ML models trained on the silver standard set. The Dx-code-count refers to the rule of predicting the disease if the diagnosis code for the disease is present in the health record

Figure 4 and Table 2 show that RF model trained on silver standard is superior or equivalent in performance to the RF model trained on the gold standard for five of the six diseases (i.e. not depression). Similarly, the LR model trained on the silver standard is superior or equivalent in performance to the LR model trained on gold standard for five of the six diseases (i.e. not breast cancer).

**Table 2.** Table of the AUROC algorithm

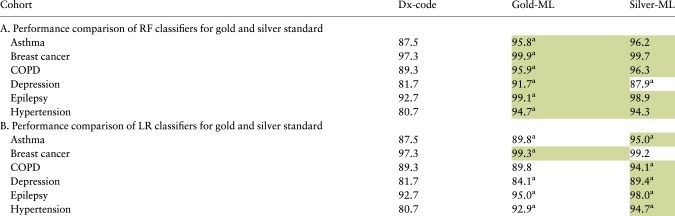

a Indicates that the value is significantly different from that in the column to the immediate left. Background green shading indicates the algorithm with the highest AUROC for the cohort.

## 4 Discussion

Our results show that the performance of ML models trained on silver standard created using PL are superior or equivalent to those trained on gold standard created by clinical experts in five of the six cohorts, for both RF and LR classifiers. The reason for the superior performance is likely due to the large size of training sets (*n* = 8000) yielded by PL, as compared to the training set of size approximately 263 from human labeling.

The latter size of 263 is because the size of the gold standard for each cohort has average size 525 (range 507–536), after excluding the uncertain categories (Table 1). As 50% of this set was used for generating the test set, the development set was restricted to (50% of 525=) 263 patients. In contrast, a total of 37 763 patients (38 026 total − 263 human labeled) were available for PL, from which training set of size 8000 could be consistently constructed for all cohorts using a polar cut-off parameter of 0.5.

The advantage of PL is that it is an intuitive approach that is easy to implement. It can be easily applied to coded data to generate silver standard. Moreover, the use of billing codes is pervasive throughout the healthcare systems across the world. However, a potential downside to PL is that the developed models maybe insensitive to newly diagnosed patients, as the polar set is inherently biased towards patients that have large number of repeated encounters with the same diagnosis. In our dataset, the count of events per patient averaged at 1330 with standard deviation of 1902. Further research is necessary to investigate the effect of data density on performance of PL. Our implementation code in the Github repository will facilitate the application of the algorithm on other datasets by the research community. To ensure reproducibility of the study, we applied PL to identify stroke patients as described in Supplementary Appendix D.

PL may be applied in situations where data points have been labeled multiple times in a noisy/biased manner. For example, in the case of healthcare data, one or more care providers evaluate a patient multiple times for presence of a disease, with each instance recorded indirectly in form of the billing codes. Hence, crowd sourcing experiments that have multiple but biased or noisy reviewers for each data point may be able to utilize PL.

The PL approach requires two parameters: the size of training set and the cut-off factor. Higher cut-off factor restricts the size of possible training set. This is because the higher cut-off pole is shifted more to the right with higher cut-off values, so there are lesser samples left available to choose from as the right-side tail diminishes.

**Fig. 5.**
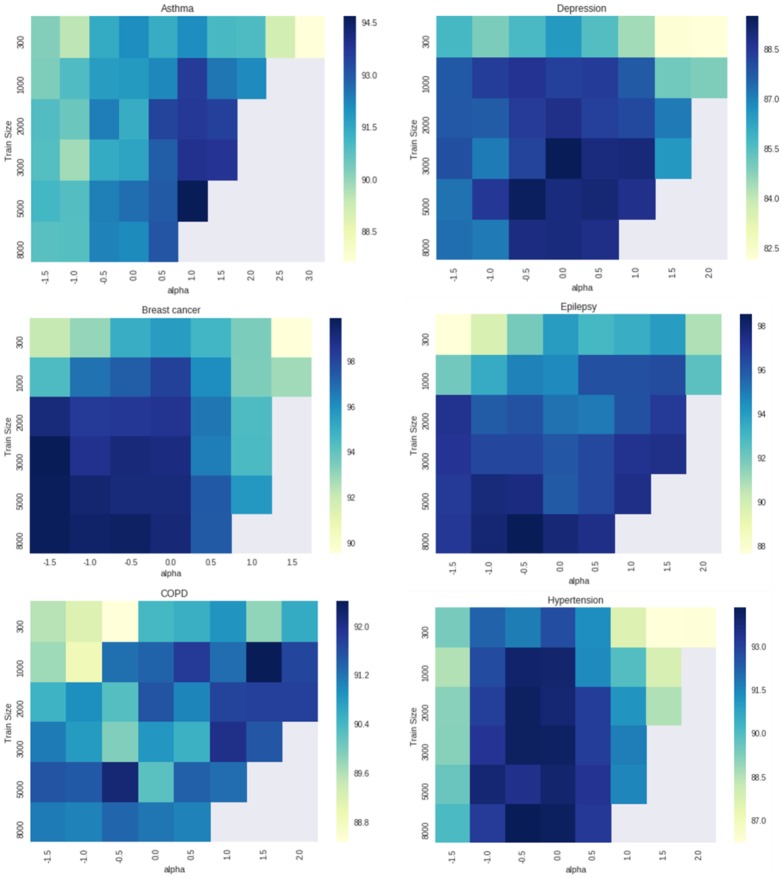
Sensitivity analysis was performed by varying ‘train size’ and ‘cut-off/alpha’ parameters for PL to create silver standard. LR models were trained on the silver standard and the performance of the models was measured by computing AUC_ROC on labeled data. The plots show that performance of ML models trained on the silver standard generally increases with higher train size

We carried out the following experiment to examine the sensitivity of the algorithm to different parameter values. We varied the training size from 300 to 8000, and the cut-off factor from −3.0 to +3.0 in 0.5 increments to generate silver standard using the unlabeled data. LR models were trained on the silver standard. The performance of the models was measured by computing AUC_ROC for predictions made on the labeled data. Figure 5 shows the variation in AUROC for the different combinations of the PL parameters (averaged over five runs). Generally higher training size improved performance of resulting ML models. The optimal cut-off parameter lies between −0.5 and 0.5, for most of the disease cohorts.

### 4.1 Limitations

A major limitation of our study is that the features selected for our datasets were based on expert judgment, which limits the generalization of our results. For high-throughput phenotyping, it is desirable to have complete automation for phenotyping and the need to engage with clinical experts for feature selection is a limitation.

In future work, we plan to expand our study dataset to include all the EHR data and to leverage automated methods for feature selection.

A second limitation of our study is that only a single clinical expert performed the annotations. In the absence of additional annotators, the reliability of the clinical expert could not be assessed.

The third limitation of the study is that we only experimented with LR and RF models and did not utilize other ML algorithms like naïve Bayes, support vector machines, neural networks, etc. Such a comparison is necessary to establish the use of PL to augmenting other ML approaches. One challenge in using the other ML algorithms is their sensitivity to data normalization. As our current PL implementation requires absolute ICD counts, we could not normalize/scale the data to evaluate other ML approaches. In future work, we will modify our implementation to allow the use of normalized datasets.

## 5 Conclusion

We have described a PL approach to create silver standard labels for training ML algorithms for disease classification. Our results demonstrate that ML models trained on silver standard created using PL are similar in performance to those trained on expert labeled data. Directions for future work include replication of the results on other cohorts and using a diversity of other ML approaches.

## Author contributions

S.N.M. and K.B.W. envisioned this study. K.B.W. developed and implemented the algorithm and carried out the experiments. H.E. provided inputs on the study design. M.K.M. performed chart review to annotate the dataset. All authors made significant contributions for drafting the manuscript and approved the final version.

## Funding

This study was supported by National Institute of Health (NIH) (R01-HG009174 and R00-LM011575).

*Conflict of Interest*: none declared.

## Supplementary Material

btaa088_Supplementary_Data
