# Supplementary material for: Polar labeling: silver standard algorithm for training disease classifiers
Source: Bioinformatics. 2020 Feb 12;36(10):3200–6. doi: 10.1093/bioinformatics/btaa088 (PMC7214041; doi:10.1093/bioinformatics/btaa088)
Supplement: btaa088_Supplementary_Data [file btaa088_supplementary_data.zip › btaa088-Suppl_Data/AppendixD_stroke_cohort.pdf]

Appendix D. Identifying cohort of stroke patients using polar labeling

To assess the ease of use and reproducibility of the study, we applied the PL algorithm for identifying patient having ischemic stroke. From the study dataset we annotated a sample of 540 patients using the methodology described in Section 2.2 of the paper (tables 2 and 3). We created features using relevant concepts hand-picked by clinical experts (table 4). Next we compared the performance of ML models trained on silver standard created using PL with those trained on the gold standard as (section 2.7 of paper).

Results presented in table 5, indicate the performance of logistic regression models trained on silver standard created using PL are superior to those trained on gold standard, while their performance of random forest models trained similarly are equivalent. These results are in agreement with the findings for main study on the six diseases reported in the paper and demonstrate that the published methodology and codebase facilitates reproducibility for other diseases.

Table 1. Prevalence of stroke

**Estimated prevalence in the general adult US population**	**Citation for Prevalence**
3 percent	Ovbiagele, B.,et. al. Stroke Epidemiology: Advancing Our Understanding of Disease Mechanism and Therapy. Neutotherapeutics 8(3) 319-329, 2011

Table 2. Annotation Criteria

Definite Criteria	Diagnosis of ischemic stroke supported by CT or MRI radiology report and no mention of a rule out diagnosis
Possible Criteria	Diagnosis of ischemic stroke with mention of confirmatory imaging but without radiological report in record and no mention of a rule out diagnosis
Not Criteria	Mention of stroke in record without mention of confirmatory imaging or no mention of stroke
Rule out diagnosis	Sah, hemorrhagic stroke, aneurysm, cerebral hemorrhage

Table 3. Distribution of expert annotations for the cohorts.
Y: present, N: Absent, P: possible, U: unknown/can't say.

	Expert annotations				
**Cohort**	**N**	**P**	**U**	**Y**	**Total**
Stroke	490	10	3	37	540

Table 4. List of variables

patient_ethnicity_Hispanic	Stroke_COD_DX_Cerebralaneurysm	Stroke_NLP_bloodclot
patient_ethnicity_Non-Hispanic	Stroke_COD_DX_Cerebraledema	Stroke_NLP_decreasedbloodpressure
patient_gender_F	Stroke_COD_DX_Fall	Stroke_NLP_edema
patient_gender_M	Stroke_COD_DX_Hypotension	Stroke_NLP_gait
patient_gender_U	Stroke_COD_DX_Ischemicheartdisease	Stroke_NLP_infarction
patient_race_Asian	Stroke_COD_DX_IschemicStroke	Stroke_NLP_infection
patient_race_Black	Stroke_COD_DX_Syncope	Stroke_NLP_lead
patient_race_Other	Stroke_COD_DX_Transientischemicattack	Stroke_NLP_magneticresonanceangiography
patient_race_Unknown	Stroke_COD_MED_Anticoagulants	Stroke_NLP_transientischaemicattack
patient_race_White	Stroke_COD_MED_Antiplateletagents	Stroke_NLP_weakness
patient_current_age	Stroke_COD_PRC_MRAngiography	Stroke_NLP_IschemicStroke
patient_dx	Stroke_NLP_aneurysms	Stroke_NLP_HemorrhagicStroke
patient_dxenct	Stroke_NLP_anticoagulant	Stroke_COD_DX_HemorrhagicStroke
patient_notes	Stroke_NLP_antiplateletagents	Stroke_COD_DX_AVM

Table 5. Table of the AUROC algorithm. * indicates that the value is significantly different from that in the column to the immediate left. Background green shading indicates the algorithm with the highest AUROC for the cohort.

A. Performance comparison of LR Classifiers for Gold and Silver standard

Disease	Dx-Code	Gold-ML	Silver-ML
Stroke	0.916	0.839*	0.979*

B. Performance comparison of RF Classifiers for Gold and Silver standard

Disease	Dx-Code	Gold-ML	Silver-ML
Stroke	0.916	0.973*	0.961
