# Supplementary material for: Polar labeling: silver standard algorithm for training disease classifiers
Source: Bioinformatics. 2020 Feb 12;36(10):3200–6. doi: 10.1093/bioinformatics/btaa088 (PMC7214041; doi:10.1093/bioinformatics/btaa088)
Supplement: btaa088_Supplementary_Data [file btaa088_supplementary_data.zip › btaa088-Suppl_Data/AppendixA_prevalence_citations.pdf]

Appendix A

Cohort	Estimated prevalence in the general adult US population (percent)	Citation for Prevalence
Asthma	7.9	Most Recent National Asthma Data: Centers for Disease Control and Prevention (CDC); 2019. Available from: https://www.cdc.gov/asthma/most_recent_national_asthma_data.htm.
Breast cancer	0.5	U.S. Cancer Statistics Working Group. U.S. Cancer Statistics Data Visualizations Tool, based on November 2018 submission data (1999–2016): U.S. Department of Health and Human Services, Centers for Disease Control and Prevention and National Cancer Institute; www.cdc.gov/cancer/dataviz, June 2019.
COPD	4 to 9	CDC Behavioral Risk Factor Surveillance System (BRFSS), 2014. COPD based on an affirmative response to the question, "Has a doctor, nurse or other health professional ever told you that you have COPD, emphysema, or chronic bronchitis?" https://www.cdc.gov
Depression	8.1	Brody DJ, Pratt LA, Hughes J. Prevalence of depression among adults aged 20 and over: United States, 2013–2016. NCHS Data Brief, no 303. Hyattsville, MD: National Center for Health Statistics. 2018.https://www.cdc.gov/nchs/products/databriefs/db303.htm
Epilepsy	1.2	National Center for Chronic Disease Prevention and Health Promotion , Division of Population Health. https://www.cdc.gov/epilepsy/data/index.html
Hypertension	29	Fryar CD, Ostchega Y, Hales CM, Zhang G, Kruszon-Moran D. Hypertension prevalence and control among adults: United States, 2015–2016. NCHS data brief, no 289. Hyattsville, MD: National Center for Health Statistics. 2017. https://www.cdc.gov/nchs/products/
