# Supplementary material for: Polar labeling: silver standard algorithm for training disease classifiers
Source: Bioinformatics. 2020 Feb 12;36(10):3200–6. doi: 10.1093/bioinformatics/btaa088 (PMC7214041; doi:10.1093/bioinformatics/btaa088)
Supplement: btaa088_Supplementary_Data [file btaa088_supplementary_data.zip › btaa088-Suppl_Data/AppendixB_annotation_criteria.pdf]

Appendix B

Disease	Definite Criteria	Possible Criteria	Not Criteria	Cautionary Diagnosis	Rule Out Diagnosis	Reference
**Asthma**	**Children<5 years old**<br>Mention of asthma in the note AND evidence of pt being on asthma medication AND asthma diagnosis remains after mention of any cautionary diagnosis<br>**Children>5 years old and Adults**<br>Mention of asthma in notes AND (evidence of asthma medication OR PFT's supporting asthma diagnosis) AND asthma diagnosis remains after mention of any cautionary diagnosis	**Children<5 years old**<br>Diagnosis of asthma without mention of patient being on asthma medication<br><br>**Children > 5 years old and Adults**<br>Mention of asthma in note without mention of patient being on asthma medication or PFT's supporting asthma diagnosis	No mention of asthma in note	CF, enlarged lymph nodes, allergic rhinosinusitis, foreign body, heart disease, tumor, viral bronchiolitis, vocal cord dysfunction, COPD, CHF, PE, mechanical airway obstruction, cough secondary to ACE inhibitors		www.nhlbi.nih.gov (NIH, NAEPP)
**Breast cancer**	Mention of breast cancer diagnosis AND evidence of breast biopsy pathology report positive for breast cancer	Mention of breast cancer diagnosis in record	No mention of breast cancer diagnosis in note			www.cancer.gov (National Cancer Institute at the NIH)
**Chronic airway obstruction (COPD)**	Mention of COPD, emphysema, chronic bronchitis, refractory asthma diagnosis AND lung function tests or spirometry test supporting copd diagnosis	Mention of COPD, emphysema, chronic bronchitis, refractory asthma diagnosis without mention of lung function test or spirometry results	No mention of COPD, emphysema, chronic bronchitis or refractory asthma			copdfoundation.org
**Depression/MDD**	2 or more mentions of depression diagnosis occurring > than 2 weeks apartANDmention of antidepressant prescribed OR psychotherapy initiated OR ECT treatmentANDno mention of a r/o diagnosis	2 or more mentions of depression diagnosis occurring > than 2 weeks apartANDno mention of a rule out diagnosis	No mention of Depression or only 1 mention of depression	Bipolar, Postpartum Depression, Schizophrenia, Schizo-affective Disorder or Mania		www.nami.org/depression
**Epilepsy**	Diagnosis of epilepsy in record AND patient on anti seizure medication AND no mention of a rule out diagnosis AND epilepsy diagnosis after mention of a cautionary diagnosis	Diagnosis of epilepsy in record	No mention of epilepsy in record	Pseudoseizures, febrile seizures, single seizure, seizures related to eclampsia	Secondary seizures related to a medical cause	www.epilepsy.com
**Hypertension**	Diagnosis of hypertension in note AND documented use of anti-hypertensives or other bp lowering regime (decrease/quit smoking, dietary changes, weight loss) AND no mention of a rule out diagnosis	2 or more mentions of hypertension diagnosis with recorded bp > 160/90 at least 3 months apart and no mention of a rule out diagnosis	Singular or no mention of hypertension or 2 mentions of hypertension less than 3 months apart only	pre-eclampsia, medication induced hypertension, hypertension due to kidney disease, diabetic neuropathy, polycystic kidney disease, glomerular disease, renovascular hypertension, cushing syndrome, coarctation of the aorta		www.mayoclinic.org
