# Supplementary material for: Polar labeling: silver standard algorithm for training disease classifiers
Source: Bioinformatics. 2020 Feb 12;36(10):3200–6. doi: 10.1093/bioinformatics/btaa088 (PMC7214041; doi:10.1093/bioinformatics/btaa088)
Supplement: btaa088_Supplementary_Data [file btaa088_supplementary_data.zip › btaa088-Suppl_Data/AppendixC_patient_features.pdf]

Appendix C Patient_features

patient_gender_F
patient_gender_M
patient_gender_U
patient_ethnicity_Hispanic
patient_ethnicity_Non-Hispanic
patient_race_Asian
patient_race_Black
patient_race_Other
patient_race_Unknown
patient_race_White
patient_current_age
patient_dx
patient_dxenct
patient_notes
Asthma_COD_DX_Allergicrhinitis
Asthma_COD_DX_Asthma
Asthma_COD_DX_Cough
Asthma_COD_DX_Emphysema
Asthma_COD_DX_Osteoporosis
Asthma_COD_MED_Bronchodilators
Asthma_COD_MED_Heparins
Asthma_COD_MED_leukotrienemodifiers
Asthma_COD_MED_Nonsteroidalantiinflammatoryagents
Asthma_COD_MED_Respiratoryinhalantproducts
Asthma_COD_PRC_AbnormalPFT
Asthma_COD_PRC_ERVisit
Asthma_COD_PRC_Inhalationtreatment
Asthma_NLP_adrenergicagonists
Asthma_NLP_allergicrhinitis
Asthma_NLP_antiinflammatoryagents
Asthma_NLP_asthma
Asthma_NLP_bronchodilators
Asthma_NLP_cough
Asthma_NLP_emergencydepartment
Asthma_NLP_emphysema
Asthma_NLP_fatigue
Asthma_NLP_fev1
Asthma_NLP_heparin
Asthma_NLP_inhaler
Asthma_NLP_leukotrienereceptorantagonist
Asthma_NLP_osteoporosis
Asthma_NLP_oxygenadministration
Asthma_NLP_pharmacotherapy

Asthma_NLP_salbutamol
Asthma_NLP_salmeterol
Asthma_NLP_seasonalallergicrhinitis
Asthma_NLP_spray
Asthma_NLP_tiotropium
Asthma_NLP_wheezing
BrCa_COD_DX_Breastcancer
BrCa_COD_DX_Intraductalcarcinoma
BrCa_COD_LAB_EGFR
BrCa_COD_PRC_Mammography
BrCa_COD_PRC_Mastectomy
BrCa_NLP_breastcancer
BrCa_NLP_breastcarcinoma
BrCa_NLP_coreneedlebiopsy
BrCa_NLP_humanepidermalgrowthfactorreceptor2
BrCa_NLP_intraductalcarcinoma
BrCa_NLP_mammography
BrCa_NLP_mastectomyprocedures
BrCa_NLP_obesity
BrCa_NLP_partialmastectomy
COPD_COD_DX_Acutebronchitis
COPD_COD_DX_Chronicischemicheartdisease
COPD_COD_DX_COPD
COPD_COD_DX_Diabetes
COPD_COD_DX_Heartfailure
COPD_COD_DX_Nicotinedependence
COPD_COD_DX_Pneumonia
COPD_COD_DX_Respiratorydistress
COPD_COD_DX_Shortnessofbreath
COPD_COD_MED_albuterol
COPD_NLP_anticholinergicagent
COPD_NLP_bodymassindex
COPD_NLP_chronicobstructivepulmonarydisease
COPD_NLP_coronaryarterydisease
COPD_NLP_decision
COPD_NLP_disability
COPD_NLP_exercise
COPD_NLP_females
COPD_NLP_fluticasone
COPD_NLP_heartfailure
COPD_NLP_highbloodpressuredisorder
COPD_NLP_influenzavaccine
COPD_NLP_lungfunction
COPD_NLP_males
COPD_NLP_nausea

COPD_NLP_oxygensaturation
COPD_NLP_smokinghistory
COPD_NLP_stroke
COPD_NLP_thought
COPD_NLP_tobacco
Depression_COD_DX_Adultandchildabuse
Depression_COD_DX_Behavioraldisorder
Depression_COD_DX_Bipolardisorder
Depression_COD_DX_DementiaorAlzheimers
Depression_COD_DX_Depression
Depression_COD_DX_Eatingdisorder
Depression_COD_DX_MDD
Depression_COD_DX_Mentalhealthdisorders
Depression_COD_DX_Psychoticdisorder
Depression_COD_DX_Substanceabuse
Depression_COD_DX_Suicideideationorattempt
Depression_COD_MED_Anticonvulsants
Depression_COD_MED_Antidepressants
Depression_COD_MED_Antipsychotics
Depression_COD_MED_MoodStabilizer
Depression_COD_PRC_ECT
Depression_NLP_abuse
Depression_NLP_antiarrhythmics
Depression_NLP_antidepressantsdrugs
Depression_NLP_depressedstate
Depression_NLP_depressivedisorders
Depression_NLP_majordepressivedisorder
Depression_NLP_mood
Depression_NLP_psychiatricdisorders
Depression_NLP_sadness
Depression_NLP_seasonaldepression
Depression_NLP_suicidalideation
Depression_NLP_transcranialmagneticstimulation
Epilepsy_COD_DX_Cerebralhemorrhage
Epilepsy_COD_DX_Convulsions
Epilepsy_COD_DX_Epilepsy
Epilepsy_COD_DX_Headinjury
Epilepsy_COD_DX_Migraine
Epilepsy_COD_MED_Benzodiazepines
Epilepsy_COD_MED_Carbamazepine
Epilepsy_COD_MED_lamotrigine
Epilepsy_COD_PRC_EEG
Epilepsy_COD_PRC_HeadCT
Epilepsy_NLP_anticonvulsants
Epilepsy_NLP_aura

Epilepsy_NLP_benzodiazepines
Epilepsy_NLP_carbamazepine
Epilepsy_NLP_clonictonicseizures
Epilepsy_NLP_ctscanning
Epilepsy_NLP_electroencephalography
Epilepsy_NLP_fainting
Epilepsy_NLP_hemorrhage
Epilepsy_NLP_injuries
Epilepsy_NLP_lamotrigine
Epilepsy_NLP_lorazepam
Epilepsy_NLP_mass
Epilepsy_NLP_phenytoin
Epilepsy_NLP_rhythm
Epilepsy_NLP_seizures
Epilepsy_NLP_seizuressyndrome
Epilepsy_NLP_shift
Epilepsy_NLP_temporallobe
HTN_COD_DX_Hypertension
HTN_COD_LAB_LDLlab
HTN_COD_MED_Aceinhibitor
HTN_COD_MED_AngiotensinIIinhibitors
HTN_COD_MED_Antihypertensives
HTN_COD_MED_Betaadrenergicblockers
HTN_COD_MED_Calciumchannelblockers
HTN_COD_MED_Thiazidediuretics
HTN_COD_PRC_Bloodpressuremonitoring
HTN_NLP_aceinhibitor
HTN_NLP_amlodipine
HTN_NLP_angiotensiniireceptorantagonists
HTN_NLP_antihypertensiveagents
HTN_NLP_atenolol
HTN_NLP_bloodpressures
HTN_NLP_dihydropyridines
HTN_NLP_hydrochlorothiazide
HTN_NLP_lisinopril
HTN_NLP_lowdensitylipoprotein
HTN_NLP_protein
HTN_NLP_thiazidediuretic
HTN_NLP_tumor
HTN_NLP_urinalysis
Asthma_COD_DX_AgeFirstAsthmaDx
COPD_COD_DX_AgeFirstCOPDDx
